# FAT2 inhibits breast cancer cell migration, invasion, and epithelial–mesenchymal transition through transcriptional upregulation of CLDN19

**DOI:** 10.3389/fonc.2026.1734983

**Published:** 2026-04-13

**Authors:** Gangyin Xie, Dengwei Lu, Ziwei Li, Tao Xu

**Affiliations:** 1 Department of General Surgery, Yunyang County People’s Hospital, Chongqing, China; 2 Department of Breast, Thyroid and Vascular Surgery, Chongqing University Fuling Hospital, School of Medicine, Chongqing University, Chongqing, China; 3 Department of Laboratory Medicine, Chongqing University Fuling Hospital, School of Medicine, Chongqing University, Chongqing, China

**Keywords:** breast cancer, CLDN19, epithelial–mesenchymal transition, FAT2, metastasis, tight junction

## Abstract

**Background:**

FAT atypical cadherin 2 (FAT2), a member of the protocadherin superfamily involved in cell adhesion and polarity, remains incompletely characterized in breast cancer. This study aimed to elucidate the expression pattern, clinical significance, and functional mechanisms of FAT2 in breast cancer.

**Methods:**

FAT2 expression and clinicopathological associations were analyzed using The Cancer Genome Atlas (TCGA) dataset. Recombinant FAT2 protein was generated, and its effects on migration, invasion, and epithelial–mesenchymal transition (EMT) were assessed in HER2-positive breast cancer cell lines (BT-474 and MDA-MB-453). Downstream effectors were identified through gene set enrichment analysis (GSEA) combined with correlation analysis and validated by gain- and loss-of-function experiments. Immunohistochemical analysis of 31 paired clinical specimens was performed to corroborate the *in vitro* findings.

**Results:**

FAT2 mRNA was significantly downregulated in breast cancer tissues and was associated with advanced clinical stage, higher T stage, and negative hormone receptor status. High FAT2 expression correlated with longer overall survival, particularly in the ER-positive subgroup. Functionally, recombinant FAT2 protein inhibited cell migration and invasion at sub-cytotoxic concentrations and reversed the EMT phenotype. Integrative bioinformatics analysis identified the tight junction protein CLDN19 as a key downstream effector of FAT2, and rescue experiments demonstrated that CLDN19 knockdown markedly attenuated FAT2-mediated anti-migratory and anti-EMT effects. Immunohistochemical analysis validated the concurrent downregulation and strong positive correlation of FAT2 and CLDN19 in clinical specimens.

**Conclusions:**

FAT2 functions as a tumor suppressor in breast cancer by inhibiting migration, invasion, and EMT through transcriptional upregulation of CLDN19. The FAT2–CLDN19 axis represents a potential prognostic biomarker and therapeutic target for anti-metastatic intervention in breast cancer.

## 1 Introduction

Breast cancer is the most prevalent malignancy and a leading cause of cancer-related mortality among women worldwide (1). Its biological heterogeneity and metastatic propensity contribute to therapeutic resistance and unfavorable outcomes (2). Despite advances in early detection and multimodal treatment, tumor invasion and metastasis remain formidable clinical challenges (3). Elucidating the molecular mechanisms governing breast cancer progression and identifying key driver genes are therefore essential for developing novel therapeutic strategies (4).

Epithelial–mesenchymal transition (EMT) is a fundamental process whereby tumor cells acquire migratory and invasive capabilities (5). Dysregulation of cell adhesion molecules, particularly claudin family members, plays a pivotal role in facilitating EMT (6, 7).

Claudins are integral components of tight junctions that are essential for maintaining epithelial polarity and barrier function. Among these, claudin-19 (CLDN19) has attracted considerable interest in oncology owing to its role in regulating paracellular permeability and intercellular adhesion. Loss or downregulation of claudin family members, including CLDN19, has been commonly associated with disrupted barrier function, increased paracellular permeability, and enhanced metastatic potential across multiple cancer types (8). In breast cancer, CLDN19 is considered a putative tumor suppressor; its expression is frequently reduced compared with that in normal breast tissue, and this downregulation correlates with higher tumor grade, lymph node metastasis, and reduced patient survival. Mechanistically, CLDN19 has been reported to inhibit tumor progression by suppressing the ECM/UBE2C/Wnt/β-catenin signaling axis, thereby attenuating EMT, proliferation, and invasion (9). However, the upstream regulatory network governing CLDN19 expression in breast cancer—particularly its potential modulation by cadherins—remains poorly understood.

The FAT protein family (FAT1–4) comprises atypical cadherins characterized by large extracellular domains containing multiple cadherin repeats that mediate cell–cell adhesion and signaling (10). Physiologically, FAT proteins play crucial roles in embryonic development and tissue polarity. In cancer, these proteins are frequently dysregulated and exhibit context-dependent functions, influencing tumor cell migration, invasion, and metastasis through modulation of adhesion, cytoskeletal dynamics, and signaling pathways such as Hippo and Wnt (11).

FAT2 shares this characteristic architecture, comprising a large N-terminal extracellular region with 34 cadherin repeats, a single-pass transmembrane domain, and a cytoplasmic tail that interacts with intracellular scaffolds and signaling molecules (12). During embryonic development, FAT2 regulates planar cell polarity, collective cell migration, and tissue boundary formation. In cancer, the role of FAT2 is context-dependent. FAT2 mutation-associated gene pairs have been identified as candidate biomarkers in gastric cancer (13), and FAT2 has been shown to modulate cell migration and immune responses through cytoskeletal and chemokine pathways (14). Given the established tumor-suppressive function of CLDN19 and the context-dependent role of FAT2, we hypothesized that a functional interplay exists between these two molecules in breast cancer. Nevertheless, the expression profile, clinical relevance, and downstream pathways of FAT2 in breast cancer—especially its relationship with CLDN19—have not been systematically investigated.

The present study was designed to characterize the expression and prognostic significance of FAT2 in breast cancer. We analyzed the associations between FAT2 expression and clinicopathological parameters using public bioinformatics databases. Recombinant FAT2 protein was produced to evaluate its effects on breast cancer cell proliferation, migration, invasion, and EMT *in vitro*. Through integrative bioinformatics and experimental validation, we uncovered a novel mechanism by which FAT2 inhibits tumor progression via upregulation of CLDN19. Our findings identify the FAT2–CLDN19 axis as a potential therapeutic target for anti-metastatic intervention.

## 2 Materials and methods

### 2.1 Materials

The Cell Counting Kit-8 (CCK-8) assay kit was obtained from Dojindo Molecular Technologies, Inc. (Kumamoto, Japan). Lipofectamine™ 3000 transfection reagent and OptiMEM^®^ Reduced-Serum Medium were purchased from Thermo Fisher Scientific (Waltham, MA, USA). Transwell^®^ chambers (polycarbonate membrane, 8 μm pore, 24-well format) and Matrigel^®^ Matrix were obtained from Corning Incorporated (Corning, NY, USA). PVDF membranes were acquired from Merck KGaA (Darmstadt, Germany). ECL chemiluminescent substrate was sourced from Thermo Fisher Scientific.

The following primary antibodies were used: anti-FAT2 (Proteintech, #31490-1-AP, 1:1000), anti-CLDN19 (Thermo Fisher Scientific, #H00149461-M02, 1:500), anti-His-Tag (D3I1O) rabbit monoclonal antibody (Cell Signaling Technology [CST], #12698, 1:1000), anti-Vimentin (CST, #5741, 1:1000), anti-N-cadherin (CST, #13116, 1:1000), anti-E-cadherin (CST, #3195, 1:1000), anti-MMP9 (CST, #13667, 1:1000), anti-GAPDH (CST, #2118, 1:2000), and anti-β-actin (CST, #4967, 1:2000).

### 2.2 Bioinformatics analysis

Transcriptomic data and clinicopathological information for breast cancer and adjacent normal tissues were downloaded from the TCGA database. Following normalization and batch-effect correction using the *limma* package in R (v4.2.0), FAT2 mRNA expression levels were compared between tumor and normal tissues using the Student’s *t*-test. Associations between FAT2 expression and clinical stage or ER/HER2 status were assessed by ANOVA or *t*-test, as appropriate. Overall survival analysis was performed using the Kaplan–Meier method with the log-rank test (via the *survival* and *survminer* packages). FAT2 protein expression in breast cancer tissues was further validated using immunohistochemical data from the Human Protein Atlas (HPA) database.

### 2.3 Plasmid construction and protein purification

A cDNA fragment encoding the human FAT2 region (amino acids 4070–4349) was synthesized and cloned into the NdeI/XhoI sites of the pET-30a(+) vector, incorporating a C-terminal 6×His tag. The construct was verified by DNA sequencing and restriction enzyme digestion and subsequently transformed into *E. coli* BL21(DE3) competent cells. Protein expression was induced with 0.2 mM IPTG at 37 °C for 16 h.

Bacterial cells were harvested, resuspended in lysis buffer (50 mM Tris-HCl pH 7.5, 200 mM NaCl, 5 mM EDTA, 0.1% Triton X-100, 0.1 mM PMSF, 50 μg/mL lysozyme), and lysed by sonication. Inclusion bodies were collected by centrifugation, washed twice with wash buffer (2 M urea in lysis buffer), and solubilized in denaturing buffer (8 M urea, 50 mM Tris-HCl pH 8.0, 300 mM NaCl).

Refolding was performed by stepwise dialysis at 4 °C against buffers containing 4 M, 2 M, 1 M, and finally 0 M urea (4–6 h per step), followed by overnight dialysis in urea-free buffer (20 mM Tris-HCl pH 8.0, 150 mM NaCl). The refolded protein was purified by Ni-NTA affinity chromatography, concentrated to 0.4 mg/mL, and stored in PBS (pH 7.4) at −80 °C until use.

Protein purity (>90%) was verified by SDS-PAGE, and identity was confirmed by Western blot analysis using an anti-His antibody (expected molecular weight ~31 kDa). The endotoxin level was determined using the Limulus Amebocyte Lysate (LAL) assay and was confirmed to be <1.0 EU/μg protein.

### 2.4 Trypsin protection assay

To evaluate the internalization of recombinant His-FAT2 protein, a trypsin protection assay was performed. BT-474 and MDA-MB-453 cells were seeded in 6-well plates and grown to 80%–90% confluency.

Cells were assigned to four groups: (1) vehicle control (protein-free medium, 37 °C); (2) total binding (His-FAT2, 0.5 μg/mL in serum-free DMEM, 37 °C, 1 h; harvested directly after three ice-cold PBS washes); (3) internalization (treated as Group 2, followed by 0.25% Trypsin-EDTA digestion at 37 °C for 10 min to remove surface-bound protein; terminated with 10% FBS-containing medium); and (4) 4 °C control (pre-chilled at 4 °C for 30 min, then treated with His-FAT2 at 4 °C for 1 h, followed by the same trypsin digestion as Group 3) to confirm energy-dependent endocytosis.

Cell lysates were prepared in RIPA buffer containing protease inhibitor cocktail. Equal amounts of protein (determined by BCA assay) were resolved by SDS-PAGE, transferred onto PVDF membranes, and blocked with 5% non-fat milk in TBST. Membranes were probed with His-Tag (D3I1O) Rabbit Monoclonal Antibody (#12698, Cell Signaling Technology; 1:1000) at 4 °C overnight, followed by HRP-conjugated goat anti-rabbit IgG secondary antibody at room temperature for 1 h. Bands were detected by enhanced chemiluminescence (ECL) and quantified using ImageJ software. The signal intensity of Group 2 was set as 100% to calculate relative internalization efficiency.

### 2.5 Cell culture and treatment

Human breast cancer cell lines BT-474 (ER^+^) and MDA-MB-453 (HER2^+^) were obtained from the American Type Culture Collection (ATCC). Both cell lines were authenticated by short tandem repeat (STR) profiling and routinely tested for mycoplasma contamination. BT-474 cells were maintained in RPMI-1640 medium, and MDA-MB-453 cells were maintained in DMEM high-glucose medium, both supplemented with 10% fetal bovine serum (FBS; Gibco) and 1% penicillin–streptomycin. Cells were cultured at 37 °C in a humidified incubator with 5% CO_2_ and passaged every 2–3 days at approximately 80–90% confluence.

### 2.6 Cell viability assay (CCK-8)

Cells in the logarithmic growth phase were seeded into 96-well plates at a density of 3–5 × 10³ cells per well. After adhesion, cells were treated with recombinant FAT2 protein at the indicated concentrations (0, 0.05, 0.1, 0.25, 0.5, and 1.0 μg/mL) for 24 or 48 h. Each condition was assessed in at least five replicate wells, with appropriate blank (medium-only) and untreated control wells included. Subsequently, 10 μL of CCK-8 reagent was added to each well, and the plates were incubated at 37 °C for 2 h. Absorbance at 450 nm was measured using a microplate reader (BioTek Synergy H1).

### 2.7 Wound-healing assay

BT-474 cells were seeded in 12-well plates at a density of 5 × 10^5^ cells per well and cultured until >90% confluence was achieved. Prior to wounding, cells were serum-starved for 12 h in medium containing 0.5% FBS. A uniform scratch was created across the monolayer using a sterile 200 μL pipette tip, and detached cells were removed by gentle washing with PBS. Cells were subsequently maintained in serum-free medium supplemented with FAT2 protein (0, 0.25, or 0.5 μg/mL). Images of the same wound field were captured at 0 and 24 h using an inverted microscope (Nikon Eclipse Ts2). Wound widths at three predetermined positions per well were measured using ImageJ software (NIH). All experiments were performed in at least three independent biological replicates, and data are expressed as mean ± standard deviation (SD).

### 2.8 Quantitative Real-Time PCR (qPCR) for CLDN19 mRNA

To quantify CLDN19 mRNA expression, BT-474 and MDA-MB-453 cells were cultured in RPMI-1640 medium supplemented with 10% FBS, seeded in 6-well plates, and treated with recombinant human FAT2 protein (0, 0.25, or 0.5 μg/mL in PBS containing 0.1% BSA) or vehicle control for 24 h. Total RNA was extracted using TRIzol™ Reagent, and RNA quality was assessed spectrophotometrically (A_260_/A_280_ ≈ 1.8–2.1; A_260_/A_230_> 1.8). One microgram of total RNA was reverse-transcribed into cDNA using the PrimeScript™ RT Kit with gDNA Eraser.

Quantitative PCR was performed in 20 μL reactions containing SYBR Green Premix, gene-specific primers for β-actin (forward: 5′-TGGCACCCAGCACAATGAA-3′; reverse: 5′-CTAAGTCATAGTCCGCCTAGAAGCA-3′; 186 bp) and CLDN19 (forward: 5′-GTGGGCCTCTATGAAGGGCT-3′; reverse: 5′-ATTGGATGTGACCGTCCAGG-3′; 106 bp), both validated for specificity, together with cDNA equivalent to 20 ng of total RNA. The thermal cycling profile consisted of an initial denaturation at 95 °C for 30 s, followed by 40 cycles of 95 °C for 5 s and 60 °C for 30 s (with fluorescence acquisition), and a melt curve analysis from 65 °C to 95 °C. All reactions were performed in triplicate.

Relative CLDN19 mRNA levels were calculated using the 2^−ΔΔCt^ method, normalized to the stable expression of β-actin, and compared with the vehicle control. Data from three independent experiments are presented as mean ± SD and were analyzed by one-way ANOVA with Dunnett’s *post-hoc* test using GraphPad Prism 9; a *P*-value < 0.05 was considered statistically significant.

### 2.9 Transwell migration and invasion assays

To evaluate the effects of FAT2 and CLDN19 on cell motility, BT-474 or MDA-MB-453 cells were allocated to five experimental groups: blank control, FAT2 (0.25 and 0.5 μg/mL), si-CLDN19, and si-CLDN19 + FAT2 (0.5 μg/mL). Cells were transfected with CLDN19-specific siRNA (or negative control siRNA) using Lipofectamine™ 3000. After 24 h, the culture medium was replaced with serum-free medium containing the indicated concentrations of FAT2, and cells were incubated for an additional 24 h prior to the assay.

For the migration assay, 5 × 10^4^ cells suspended in 200 μL of serum-free medium were plated in the upper chamber of a Transwell insert (8 μm pore size). The lower chamber was filled with 600 μL of medium containing 10% FBS as a chemoattractant. For the invasion assay, the membrane was pre-coated with 50 μL of Matrigel (diluted 1:8 in serum-free medium; polymerized at 37 °C for 1 h), and 1 × 10^5^ cells were seeded per insert. After 24 h of incubation, cells remaining on the upper surface were removed with a cotton swab. Cells that had migrated or invaded through to the lower surface were fixed in 4% paraformaldehyde for 20 min, stained with 0.1% crystal violet for 30 min, and counted in five randomly selected fields under a light microscope. Each experiment was performed in triplicate and repeated independently three times.

### 2.10 Western blot analysis

Following treatment with FAT2 (0, 0.25, or 0.5 μg/mL) for 24 h, cells were lysed in RIPA buffer supplemented with protease and phosphatase inhibitor cocktails. Protein concentration was determined using the BCA assay. Equal amounts of protein (20–40 μg) were separated by 10% SDS-PAGE and transferred onto PVDF membranes. Membranes were blocked with 5% non-fat milk for 2 h at room temperature and then incubated overnight at 4 °C with the following primary antibodies: anti-FAT2 (1:1000), anti-CLDN19 (1:500), anti-Vimentin (1:1000), anti-N-cadherin (1:1000), anti-E-cadherin (1:1000), anti-MMP9 (1:1000), and anti-GAPDH (1:2000). After thorough washing, membranes were incubated with HRP-conjugated secondary antibodies (1:5000) for 1 h at room temperature. Protein bands were visualized using an ECL chemiluminescence detection system and quantified using ImageJ software.

### 2.11 siRNA-mediated gene knockdown

To knock down the expression of human CLDN19 (Ensembl: ENSG00000164007) and FAT2 (Ensembl: ENSG00000086570), three independent siRNAs targeting each gene were designed using the BLOCK-iT™ RNAi Designer tool (Thermo Fisher Scientific) to minimize potential off-target effects. A scrambled negative control siRNA (siNC) was included for both gene sets. All siRNA sequences (see **Tables 1**, **2**) were synthesized by Anhui General Biological Systems Co., Ltd.

**Table 1:** siRNA sequences used for CLDN19 knockdown.

Name	Strand	Sequence (5′→3′)
siCLDN19-1	Sense	CCGAGAACCAGUUGUUAAATT
	Antisense	UUUAACAACUGGUUCUCGGTT
siCLDN19-2	Sense	CAGGAGUUCUUCAACCCAATT
	Antisense	UUGGGUUGAAGAACUCCUGTT
siCLDN19-3	Sense	GGACGGUCACAUCCAAUCATT
	Antisense	UGAUUGGAUGUGACCGUCCTT
siNC	Sense	UUCUCCGAACGUGUCACGUTT
	Antisense	ACGUGACACGUUCGGAGAATT

**Table 2:** siRNA sequences used for FAT2 knockdown.

Name	Strand	Sequence (5′→3′)
siFAT2-1	Sense	GAGAUGAGUGGAGGAUUUC
	Antisense	GAAAUCCUCCACUCAUCUC
siFAT2-2	Sense	CCACAAAGCUCAUGGACUU
	Antisense	AAGUCCAUGAGCUUUGUGG
siFAT2-3	Sense	GCAAUGAACUAGAGUAUUU
	Antisense	AAAUACUCUAGUUCAUUGC
siNC	Sense	UUCUCCGAACGUGUCACGU
	Antisense	ACGUGACACGUUCGGAGAA

Lyophilized siRNAs were briefly centrifuged, dissolved in nuclease-free water to a stock concentration of 20 μM, and aliquoted for storage at −20 °C. For transfection, BT-474 cells were transfected with the respective siRNAs or siNC using Lipofectamine™ 3000 according to the manufacturer’s protocol. After 48–72 h, knockdown efficiency for each target gene was validated by Western blot. The siRNA yielding the highest knockdown efficiency for each gene (siCLDN19–1 and siFAT2-1, respectively) was selected for all subsequent functional experiments.

### 2.12 Immunohistochemical analysis

Thirty-one paired breast cancer and adjacent normal tissue samples were collected with written informed consent and institutional ethics committee approval. Tissues were fixed in 4% paraformaldehyde, embedded in paraffin, and sectioned at 4 μm thickness. For IHC staining, sections were deparaffinized, rehydrated, and subjected to antigen retrieval in citrate buffer (pH 6.0) using a microwave heating method. Endogenous peroxidase activity was blocked with 3% H_2_O_2_. After blocking with 5% normal goat serum, sections were incubated overnight at 4 °C with primary antibodies against FAT2 (Proteintech, #31490-1-AP, 1:200) or CLDN19 (Thermo Fisher, #H00149461-M02, 1:150). Subsequently, sections were incubated with HRP-conjugated secondary antibodies at room temperature for 1 h, followed by visualization with 3,3′-diaminobenzidine (DAB) and counterstaining with hematoxylin. Staining intensity (0, none; 1, weak; 2, moderate; 3, strong) and the percentage of positive tumor cells were evaluated independently by two board-certified pathologists blinded to the clinical data. The final IHC score was calculated by multiplying the intensity score by the percentage of positive cells (range 0–300).

### 2.13 Statistical analysis

All statistical analyses and data visualization were performed using GraphPad Prism 9.0. Continuous data are presented as mean ± SD. Data normality was assessed using the Shapiro–Wilk test, and homogeneity of variances was confirmed using the Brown–Forsythe test. Two-group comparisons were performed using unpaired (or paired) Student’s *t*-test. Multi-group comparisons were conducted using one-way ANOVA followed by Tukey’s *post-hoc* test for pairwise comparisons when a significant overall difference was detected. Correlations between variables were evaluated using Spearman’s rank correlation coefficient. Overall survival was estimated by the Kaplan–Meier method, with patients dichotomized into high- and low-expression groups based on the median FAT2 expression level. Differences between survival curves were assessed using the log-rank test. A *P*-value < 0.05 was considered statistically significant.

## 3 Results

### 3.1 Downregulation of FAT2 expression is associated with adverse clinicopathological features and poor prognosis in breast cancer

**Figure 1:**
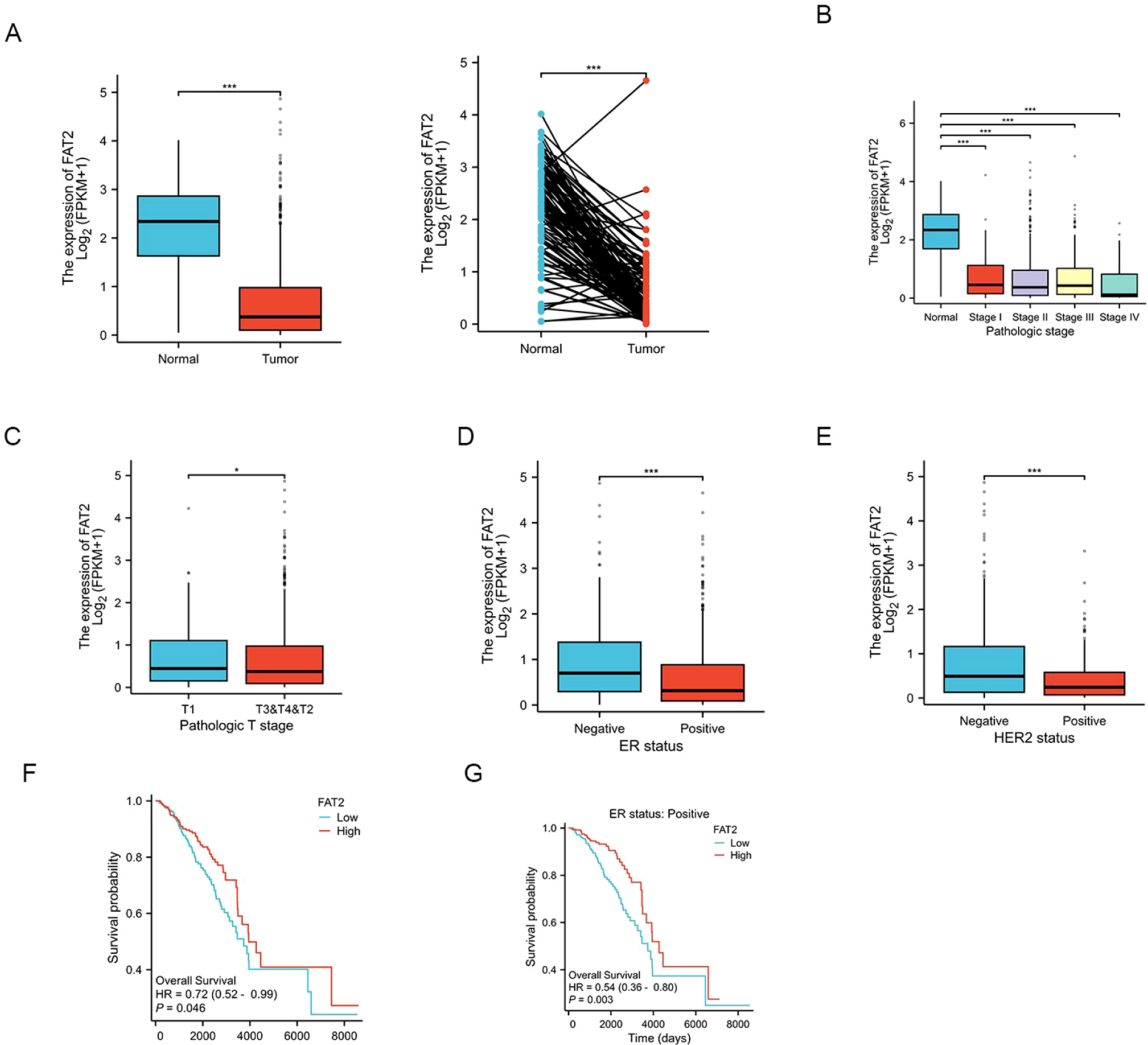
Downregulation of FAT2 expression is associated with adverse clinicopathological features and poor prognosis in breast cancer. **(A)** Comparison of FAT2 mRNA expression levels between normal breast tissues and breast cancer tissues (TCGA data). **(B, C)** Association of FAT2 expression with clinical stage **(B)** and T stage **(C)** in breast cancer patients. **(D, E)** Distribution of FAT2 expression across breast cancer subtypes defined by ER status **(D)** and HER2 status **(E)**. **(F, G)** Kaplan–Meier overall survival curves demonstrating that low FAT2 expression correlates with worse prognosis in the entire breast cancer cohort **(F)** and in the ER-positive subgroup **(G)** (TCGA data). *P < 0.05, ***P < 0.001.

Analysis of the TCGA dataset revealed that FAT2 mRNA expression was significantly downregulated in breast cancer tissues relative to paired adjacent normal tissues (*P* < 0.001, Student’s *t*-test; **Figure 1A**). Clinicopathological association analysis further demonstrated that reduced FAT2 expression was significantly associated with a more advanced clinical stage (*P* = 0.003), higher T stage (*P* = 0.012; **Figures 1B, C**), and aggressive tumor features, including negative estrogen receptor (ER) and progesterone receptor (PR) status (all *P* < 0.05; **Table 3**). Subtype-specific analysis revealed that FAT2 expression was notably lower in both ER-positive and HER2-positive breast cancer subtypes relative to their respective normal counterparts (**Figures 1D, E**). Collectively, these findings are consistent with a potential tumor-suppressive role for FAT2 in breast cancer.

**Table 3:** Clinicopathological characteristics of breast cancer patients stratified by FAT2 expression.

Characteristics	Low FAT2	High FAT2	Value
n	543	544	
Pathologic T stage, n (%)			0.012
T1	130 (12.0%)	148 (13.7%)	
T2	331 (30.5%)	300 (27.7%)	
T3	57 (5.3%)	83 (7.7%)	
T4	23 (2.1%)	12 (1.1%)	
Pathologic N stage, n (%)			0.871
N0	256 (24.0%)	260 (24.3%)	
N1	176 (16.5%)	183 (17.1%)	
N2	61 (5.7%)	55 (5.1%)	
N3	36 (3.4%)	41 (3.8%)	
Pathologic M stage, n (%)			0.368
M0	451 (48.8%)	454 (49.1%)	
M1	12 (1.3%)	8 (0.9%)	
Pathologic stage, n (%)			0.456
Stage I	85 (8.0%)	97 (9.1%)	
Stage II	317 (29.8%)	302 (28.4%)	
Stage III	116 (10.9%)	128 (12.0%)	
Stage IV	11 (1.0%)	7 (0.7%)	
PR status, n (%)			< 0.001
Negative	135 (13.1%)	207 (20.0%)	
Positive	377 (36.5%)	315 (30.5%)	
ER status, n (%)			< 0.001
Negative	73 (7.0%)	167 (16.1%)	
Positive	442 (42.6%)	355 (34.2%)	
HER2 status, n (%)			< 0.001
Negative	254 (35.4%)	306 (42.7%)	
Positive	76 (10.6%)	81 (11.3%)	

Kaplan–Meier survival analysis demonstrated that patients with high FAT2 expression (stratified by the median value) had significantly longer overall survival than those with low expression (HR = 0.72, 95% CI: 0.52–0.99; log-rank *P* = 0.046; **Figure 1F**), and this protective association was particularly pronounced in the ER-positive subgroup (**Figure 1G**). However, multivariate Cox regression analysis incorporating standard prognostic factors (age, clinical stage, and ER status) revealed that FAT2 expression did not retain independent prognostic significance (**Table 4**), suggesting that the prognostic value of FAT2 may be partially confounded by its strong association with established prognostic indicators such as ER and PR status.

**Table 4:** Univariate and multivariate Cox regression analyses of overall survival in breast cancer patients.

Variable	N	Univariate		Multivariate	
		HR (95% CI)		HR (95% CI)	
T stage					
T1	277	Reference		Reference	
T2	631	1.336 (0.890–2.006)	0.162	0.828 (0.466–1.470)	0.519
T3	140	1.551 (0.921–2.612)	0.099	1.578 (0.700–3.556)	0.272
T4	35	3.759 (1.959–7.213)	< 0.001	4.586 (1.460–14.404)	0.009
N stage					
N0	516	Reference		Reference	
N1	358	1.947 (1.322–2.865)	< 0.001	1.425 (0.819–2.479)	0.210
N2	116	2.522 (1.484–4.287)	< 0.001	1.641 (0.775–3.475)	0.196
N3	77	4.191 (2.318–7.580)	< 0.001	2.225 (0.773–6.403)	0.138
M stage					
M0	905	Reference		Reference	
M1	20	4.266 (2.474–7.354)	< 0.001	3.422 (1.323–8.849)	0.011
ER status					
Negative	240	Reference		Reference	
Positive	796	0.709 (0.493–1.019)	0.063	0.482 (0.285–0.814)	0.006
HER2 status					
Negative	560	Reference		Reference	
Positive	157	1.593 (0.973–2.609)	0.064	1.179 (0.662–2.097)	0.576
FAT2 expression					
Low	542	Reference		Reference	
High	544	0.722 (0.524–0.994)	0.046	0.694 (0.410–1.174)	0.173

### 3.2 Recombinant FAT2 protein: design, preparation, and effect on cell viability

**Figure 2:**
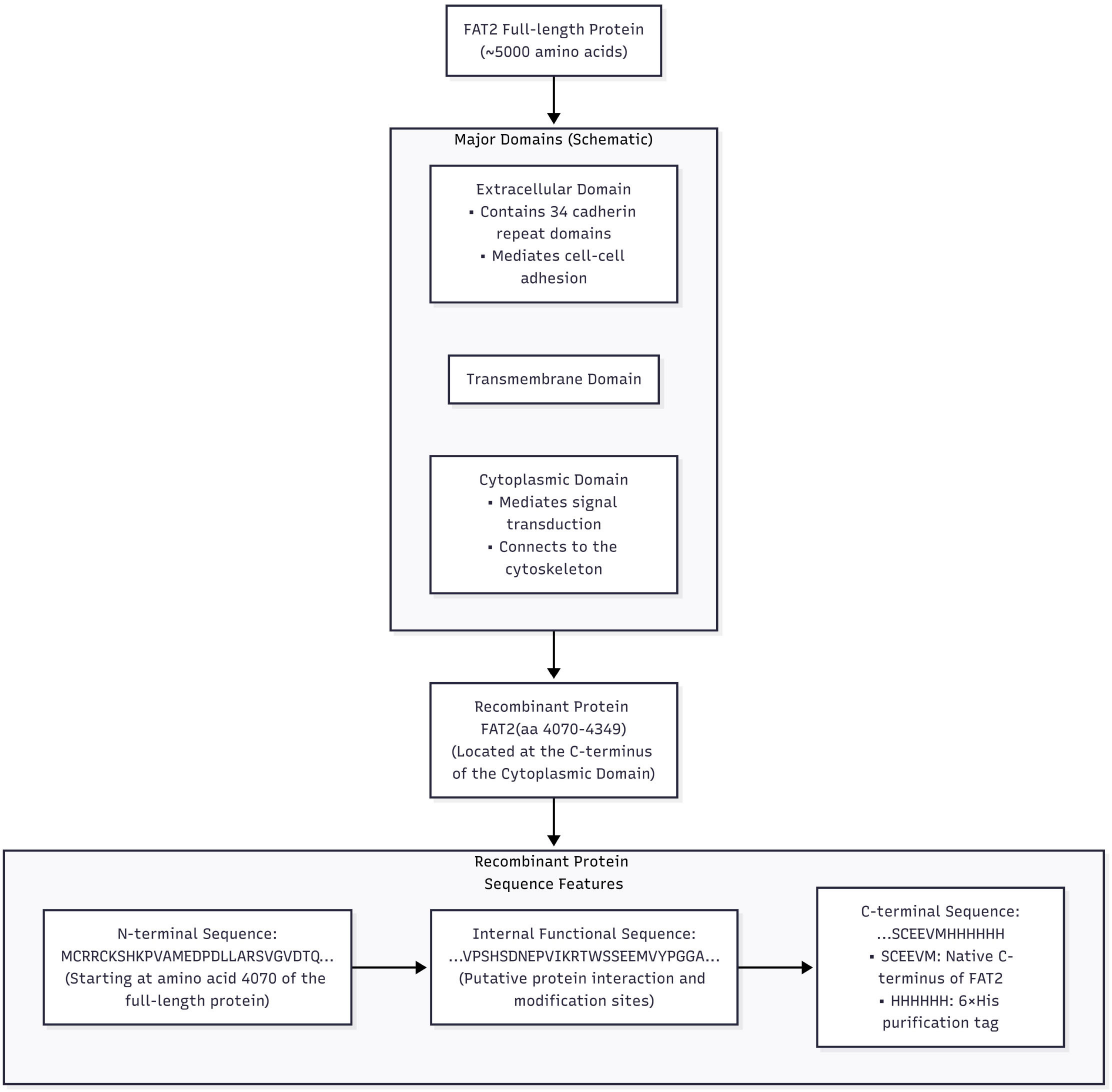
Domain architecture of FAT2 and the region used for recombinant protein construction. Schematic representation of the full-length FAT2 protein domain structure. The region selected for recombinant construct generation is highlighted, with annotations of known or putative domain functions to provide a structural rationale for the design.

The domain architecture of FAT2 and the region selected for recombinant protein production are illustrated in **Figure 2**, with annotations of the relevant functional domains that provided the structural rationale for construct design.

**Figure 3:**
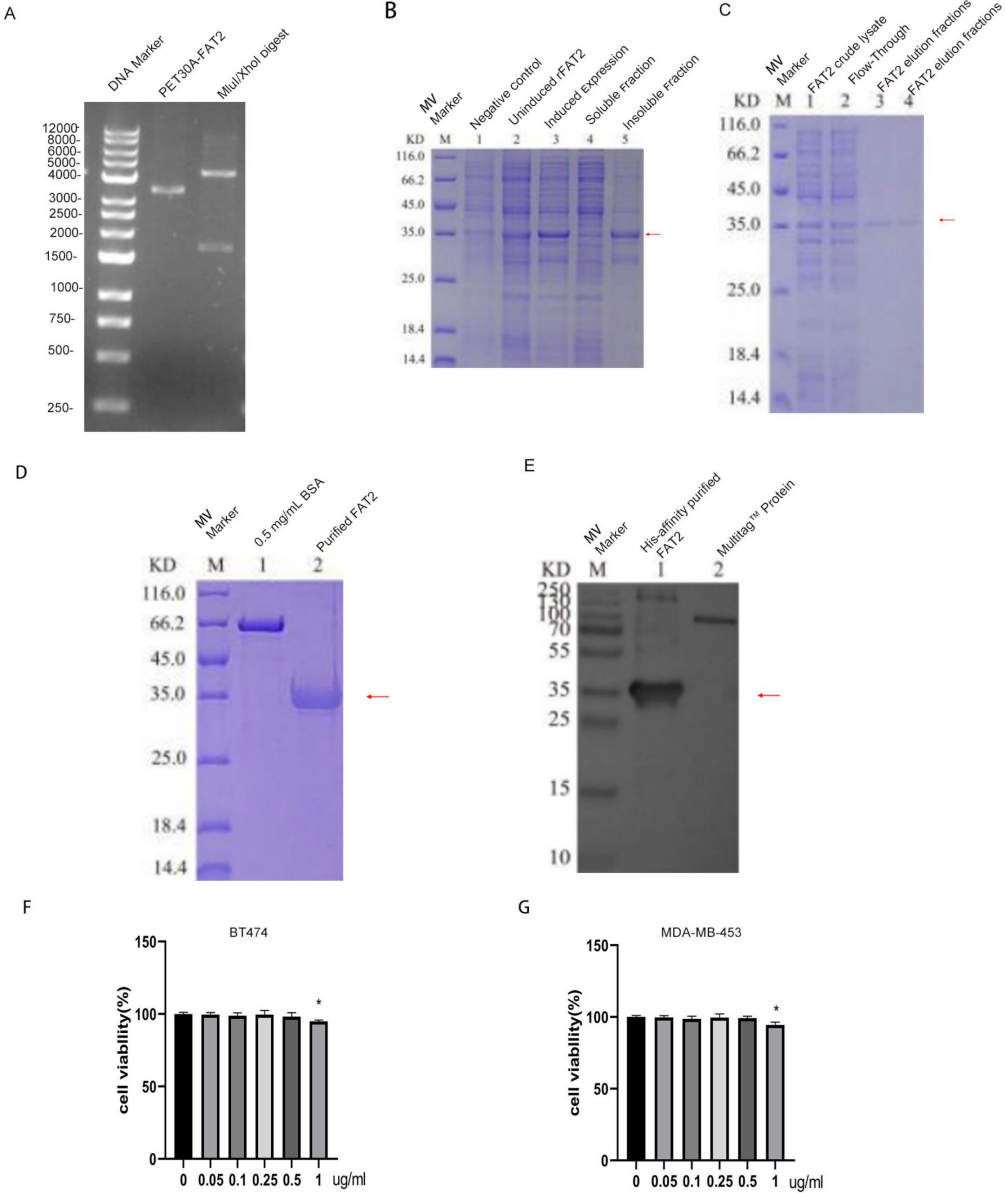
Expression, purification, and functional validation of recombinant FAT2 protein. **(A)** Agarose gel electrophoresis of the pET-30a-fat2 plasmid digested with NdeI and XhoI, confirming successful cloning. **(B)** SDS-PAGE analysis showing induced expression of recombinant FAT2 protein in *E. coli* BL21(DE3) (arrow indicates the target band). **(C)** Evaluation of the FAT2 purification process by Ni-NTA affinity chromatography. **(D)** SDS-PAGE analysis of the final purified FAT2 product. **(E)** Western blot analysis of purified FAT2 using an anti-His tag antibody, confirming protein identity. **(F, G)** CCK-8 assays showing that recombinant FAT2 protein at 1 μg/mL significantly inhibits the viability of BT-474 **(F)** and MDA-MB-453 **(G)** cells at 24 and 48 h, while lower concentrations (0.05–0.5 μg/mL) exert no significant cytotoxic effect. Data are presented as mean ± SD from at least five replicates. *P < 0.05, **P < 0.01 vs. vehicle control.

The recombinant plasmid pET-30a-fat2 was successfully constructed and verified by restriction enzyme digestion (**Figure 3A**). Upon IPTG induction in *E. coli* BL21(DE3), SDS-PAGE analysis confirmed expression of the target protein, which was predominantly present in the insoluble fraction as inclusion bodies (**Figure 3B**). The inclusion bodies were solubilized under denaturing conditions, and the protein was subsequently refolded by stepwise dialysis and purified by Ni-NTA affinity chromatography, yielding highly purified recombinant FAT2 protein (**Figures 3C, D**). The identity and specificity of the purified protein were further validated by Western blot analysis using an anti-FAT2 antibody (**Figure 3E**).

To establish an appropriate working concentration range, the effect of recombinant FAT2 on cell viability was evaluated using the CCK-8 assay. Treatment of BT-474 and MDA-MB-453 cells with FAT2 at concentrations of 0–1 μg/mL for 24 or 48 h demonstrated that only the highest concentration tested (1 μg/mL) significantly reduced cell viability (*P* < 0.01 vs. vehicle control; **Figures 3F, G**), whereas concentrations of 0.05–0.5 μg/mL had no significant effect. Based on these results, sub-cytotoxic concentrations of 0.25 and 0.5 μg/mL were selected for subsequent functional assays, thereby enabling assessment of the effects of FAT2 on cell motility independently of cytotoxicity.

### 3.3 FAT2 inhibits migration, invasion, and the epithelial–mesenchymal transition in breast cancer cells

**Figure 4:**
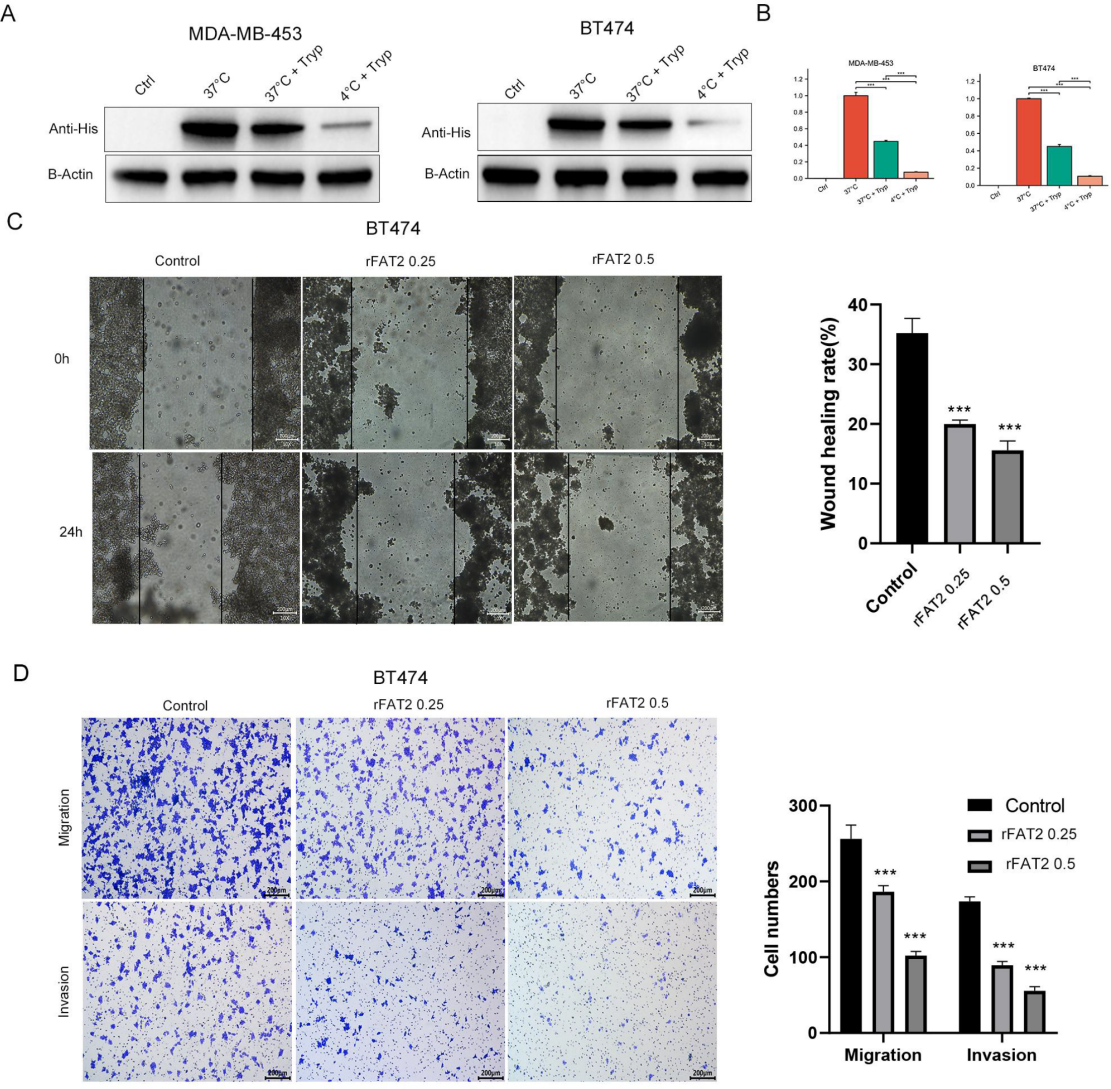
Recombinant FAT2 protein is internalized by HER2-positive breast cancer cells and suppresses migration and invasion of BT-474 cells. **(A, B)** Trypsin protection assay in BT-474 **(A)** and MDA-MB-453 **(B)** cells. Cells were incubated with His-FAT2 recombinant protein (0.5 μg/mL) and analyzed by Western blot using an anti-His antibody. Ctrl, untreated control; 37 °C, His-FAT2 incubation without trypsin; 37 °C + Tryp, His-FAT2 incubation followed by trypsin digestion at 37 °C; 4 °C + Tryp, His-FAT2 incubation followed by trypsin digestion at 4 °C. Trypsin-resistant bands indicate internalized protein. **(C)** Representative images of wound-healing assay showing the effect of recombinant FAT2 protein on BT-474 cell migration at 0 and 24 h. **(D)** Quantification of Transwell migration and Matrigel invasion assays demonstrating the inhibitory effects of FAT2 protein on BT-474 cell motility and invasiveness. Data are presented as mean ± SD from three independent experiments. **P < 0.01, ***P < 0.001 versus vehicle control.

Trypsin protection assays were conducted to assess the internalization of recombinant FAT2 protein in BT-474 and MDA-MB-453 cells (**Figures 4A, B**). No His-FAT2 signal was detected in untreated control cells, confirming assay specificity. Using the 37 °C non-trypsinized group as the reference (100%), trypsin digestion at 37 °C reduced detectable His-FAT2 to approximately 45% and 44.69% in BT-474 and MDA-MB-453 cells, respectively, indicating that approximately half of the protein was internalized and thus protected from proteolytic degradation. When endocytosis was inhibited at 4 °C, only 10.60% and 7.50% of the protein remained detectable, confirming the susceptibility of surface-bound His-FAT2 to protease cleavage. These findings demonstrate that recombinant FAT2 protein is actively internalized through an energy-dependent endocytic pathway.

**Figure 5:**
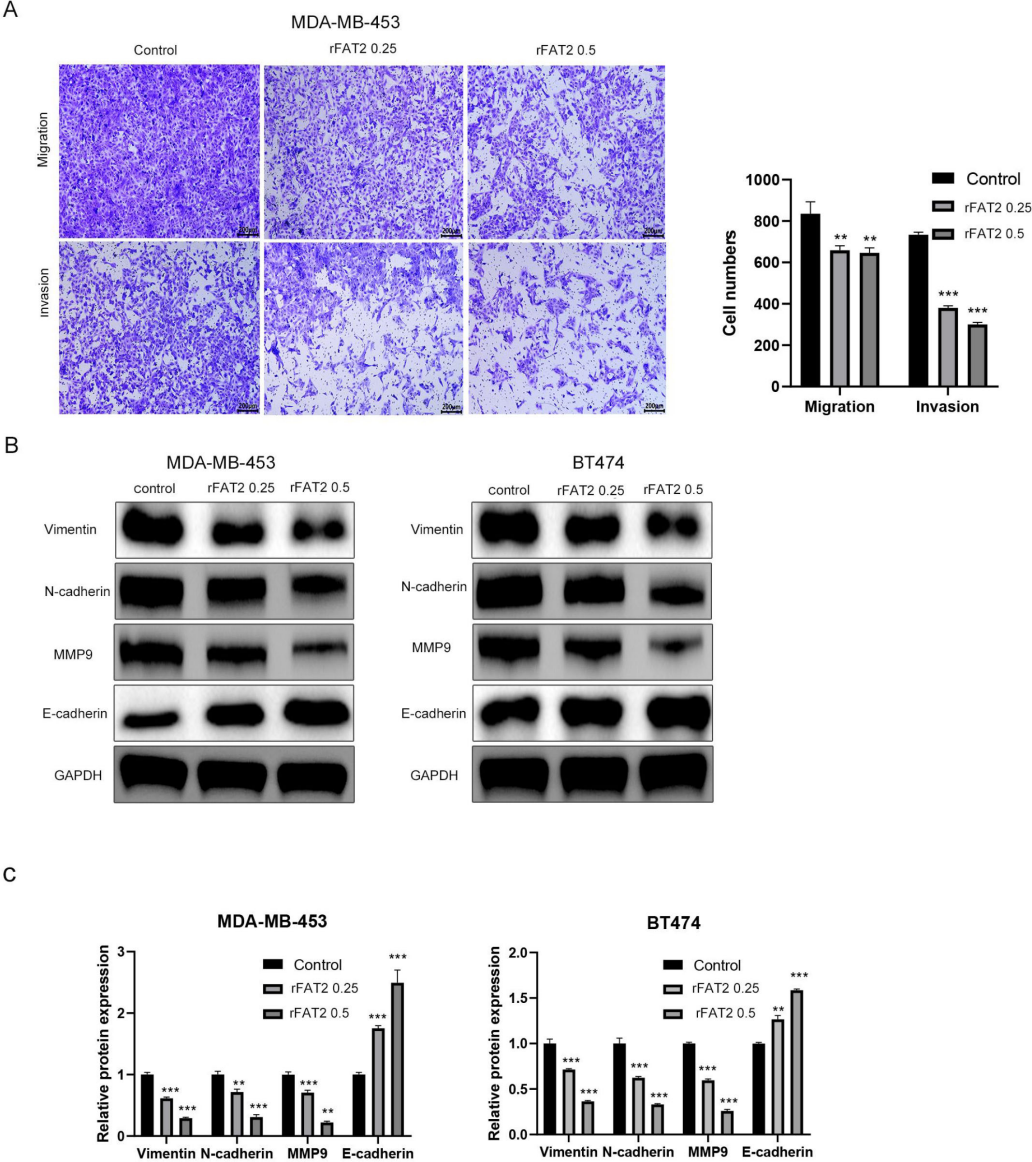
Recombinant FAT2 protein suppresses migration, invasion, and epithelial–mesenchymal transition (EMT) in breast cancer cells. **(A)** Transwell migration and invasion assays demonstrating that FAT2 treatment dose-dependently inhibits the migratory and invasive capacities of MDA-MB-453 cells. **(B, C)** Western blot analysis of EMT-related markers (E-cadherin, N-cadherin, Vimentin, and MMP9) in BT-474 **(B)** and MDA-MB-453 **(C)** cells treated with the indicated concentrations of FAT2 for 24 h. Data are presented as mean ± SD from three independent experiments. **P < 0.01, ***P < 0.001 vs. untreated control.

Functional assays further demonstrated that FAT2 significantly suppressed cell migration and invasion. In wound-healing assays, FAT2 at 0.25 and 0.5 μg/mL reduced BT-474 cell migration by 45% and 68% at 24 h, respectively (P < 0.001; **Figure 4C**). Transwell invasion assays showed that FAT2 (0.5 μg/mL) decreased invading BT-474 cells by approximately 60% (P < 0.001; **Figure 4D**). Comparable effects were observed in MDA-MB-453 cells (**Figure 5A**), indicating that these inhibitory activities are not cell-line–specific.

At the molecular level, Western blot analysis revealed that FAT2 treatment (0.5 μg/mL, 24 h) upregulated E-cadherin expression (approximately 2.0-fold) while downregulating N-cadherin, Vimentin (approximately 70% reduction), and MMP9 levels in both cell lines (all P < 0.01; **Figures 5B, C**). These data suggest that FAT2 inhibits the migration and invasion of HER2-positive breast cancer cells, at least in part, through reversal of the EMT program.

### 3.4 FAT2 exerts anti-migratory effects by upregulating CLDN19 expression

**Figure 6:**
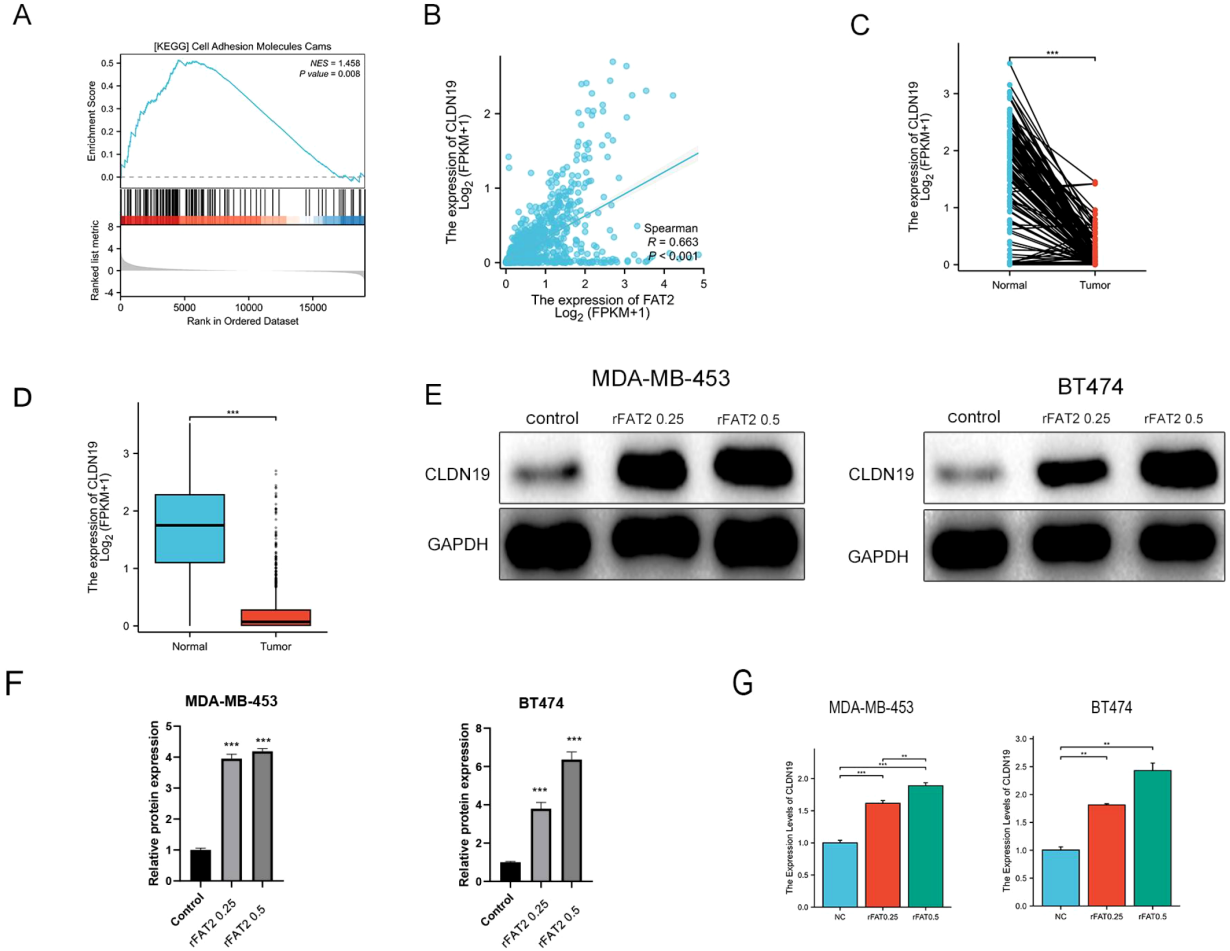
FAT2 positively regulates CLDN19 expression in breast cancer. **(A)** Gene Set Enrichment Analysis (GSEA) of FAT2-associated gene expression in the TCGA-BRCA dataset, showing significant enrichment of cell adhesion–related pathways. **(B)** Scatter plot showing the positive correlation between FAT2 and CLDN19 mRNA expression levels in breast cancer (Spearman’s *r* = 0.663, *P* < 0.001; TCGA data). **(C, D)** CLDN19 mRNA expression is significantly downregulated in breast cancer tissues compared with adjacent normal tissues (TCGA data). **(E–G)** Western blot **(E, F)** and qPCR **(G)** analyses showing dose-dependent upregulation of CLDN19 protein and mRNA levels in BT-474 and MDA-MB-453 cells treated with recombinant FAT2 protein (0, 0.25, 0.5 μg/mL) for 24 h. Data are presented as mean ± SD from three independent experiments. **P < 0.01, ***P < 0.001.

To identify downstream effectors mediating the anti-migratory function of FAT2, an integrative bioinformatics approach was employed. GSEA of TCGA-BRCA RNA-seq data stratified by FAT2 expression revealed significant enrichment of gene sets related to cell–cell adhesion in the FAT2-high group (e.g., “KEGG_Cell Adhesion Molecules,” NES = 1.458, *P* = 0.008; **Figure 6A**), indicating that FAT2 may regulate intercellular junction pathways.

Among the adhesion-related genes, CLDN19, a core component of tight junctions, emerged as the top candidate positively correlated with FAT2 expression (Spearman’s *r* = 0.663, *P* < 0.001; **Figure 6B**). Similar to FAT2, CLDN19 mRNA expression was significantly downregulated in breast tumor tissues compared with adjacent normal tissues in the TCGA dataset (**Figures 6C, D**).

**Figure 7:**
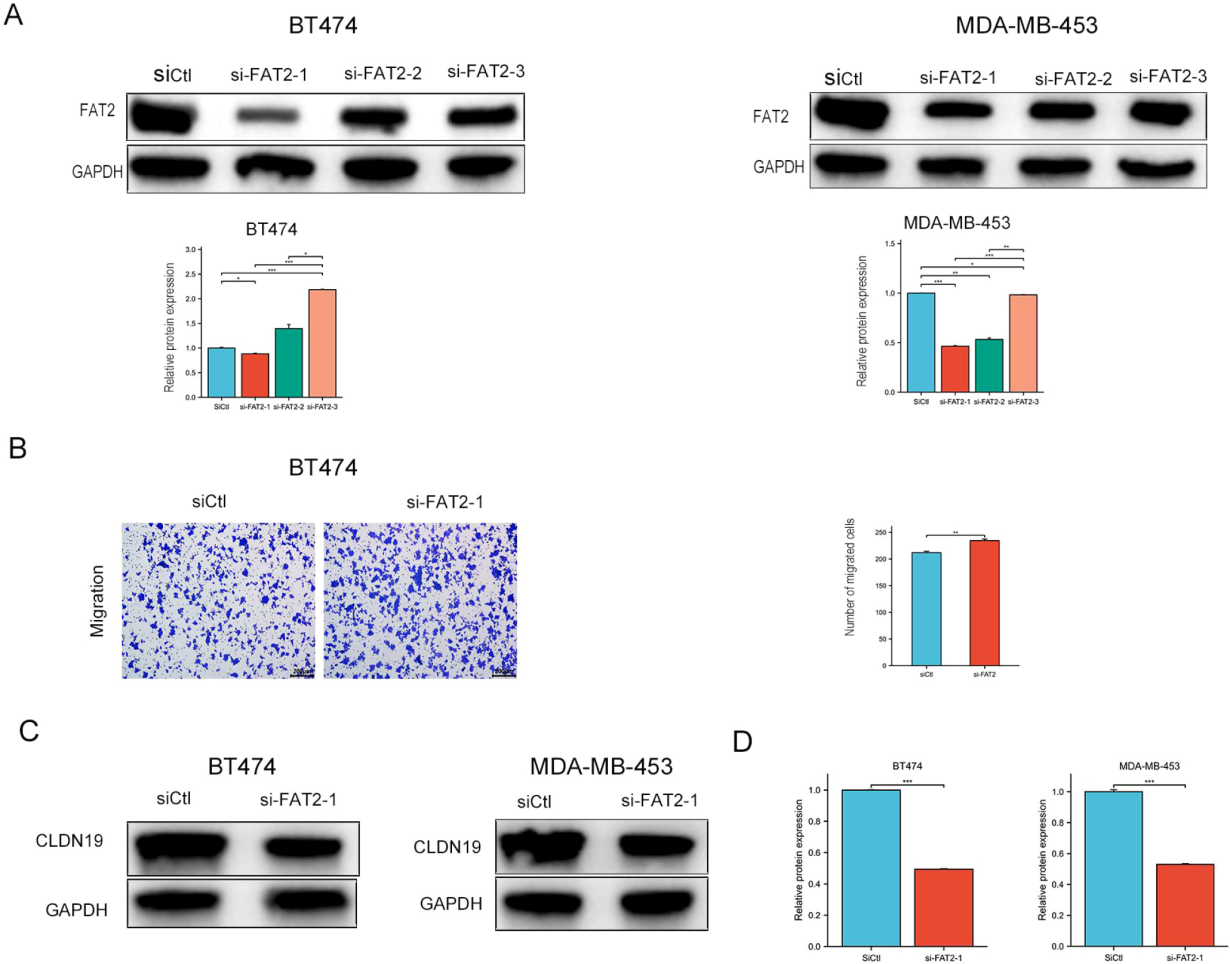
Knockdown of FAT2 promotes cell migration and downregulates CLDN19 expression. **(A)** Western blot validation of FAT2 knockdown efficiency in BT-474 and MDA-MB-453 cells transfected with FAT2-targeting siRNAs or negative control siRNA (siNC). **(B)** Transwell migration assay showing that FAT2 knockdown significantly promotes BT-474 cell migration. Quantification is shown in the right panel. **(C, D)** Western blot analysis demonstrating reduced CLDN19 protein expression following FAT2 knockdown in BT-474 **(C)** and MDA-MB-453 **(D)** cells. Data are representative of three independent experiments. *P < 0.05, **P < 0.01, ***P < 0.001 vs. siNC group.

This correlation was subsequently validated at the functional level *in vitro*. Treatment of BT-474 and MDA-MB-453 cells with recombinant FAT2 protein (0.25 and 0.5 μg/mL) for 24 h led to a dose-dependent upregulation of both CLDN19 mRNA and protein levels (**Figures 6E–G**). Conversely, siRNA-mediated knockdown of FAT2 resulted in significant downregulation of CLDN19 protein expression in both cell lines (**Figures 7A, C, D**) and correspondingly promoted cell migration (**Figure 7B**). Together, these gain- and loss-of-function data establish FAT2 as a positive upstream regulator of CLDN19.

**Figure 8:**
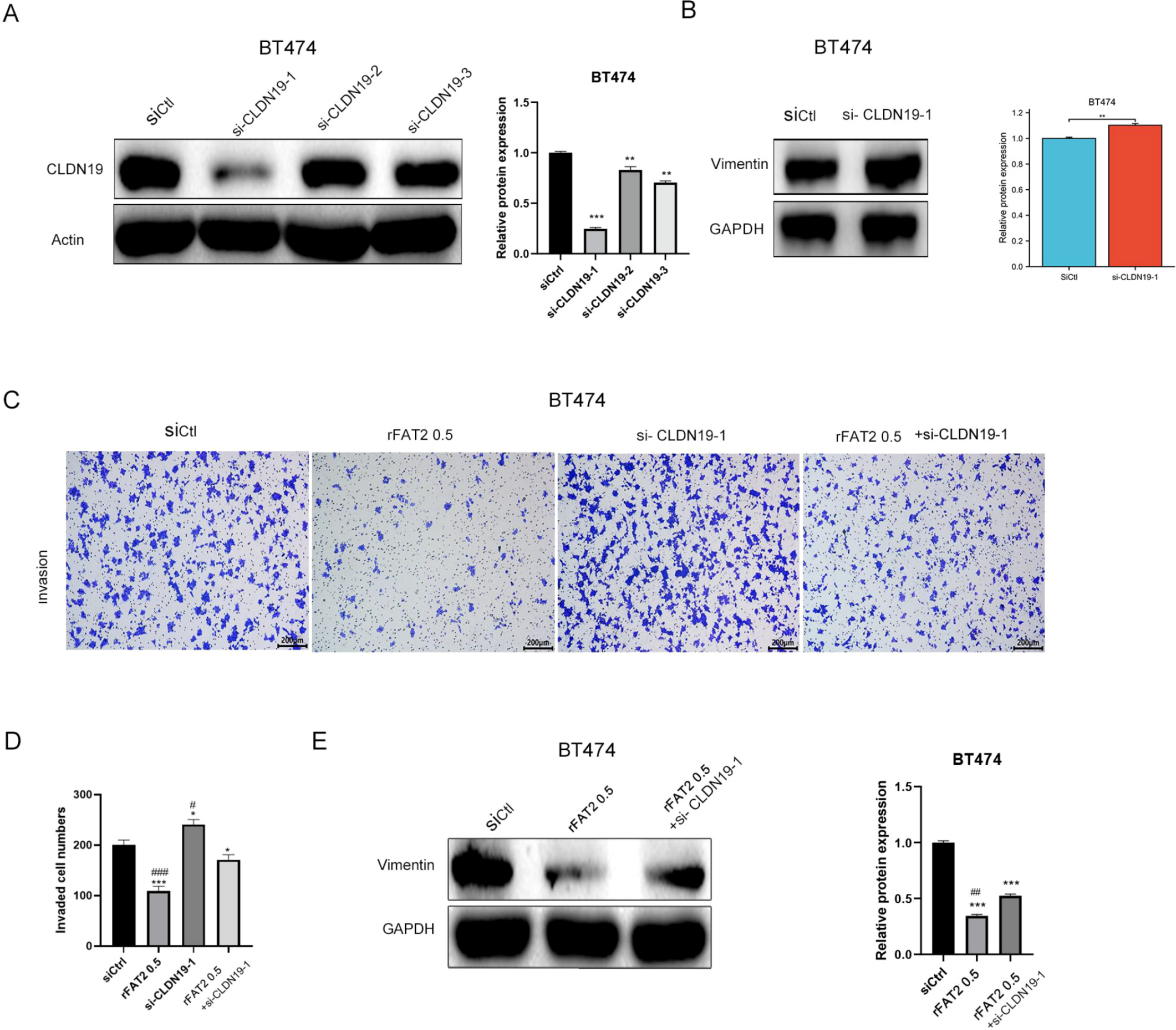
CLDN19 mediates the anti-migratory and anti-EMT effects of FAT2 in breast cancer cells. **(A)** Western blot analysis confirming CLDN19 knockdown efficiency in BT-474 cells transfected with three independent siRNAs targeting CLDN19. **(B)** Western blot analysis of Vimentin expression in BT-474 cells 48 h after transfection with siCLDN19-1. **(C, D)** Transwell migration assay showing that concurrent CLDN19 knockdown partially rescues the inhibition of BT-474 cell migration induced by recombinant FAT2 protein (0.5 μg/mL). Representative images **(C)** and quantification **(D)** are shown. **(E)** Western blot analysis demonstrating that CLDN19 knockdown partially reverses the FAT2-induced downregulation of Vimentin. Data are representative of three independent experiments and presented as mean ± SD. *P < 0.05, **P < 0.01, ***P < 0.001 vs. siNC group; ^#^*P* < 0.05, ^###^*P* < 0.001 vs. FAT2 (0.5 μg/mL) + siCLDN19–1 group.

To ascertain whether CLDN19 is a necessary mediator of the anti-migratory effect of FAT2, rescue experiments were performed. Following confirmation of efficient siRNA-mediated CLDN19 knockdown (**Figure 8A**), migration assays revealed that the inhibitory effect of recombinant FAT2 protein (0.5 μg/mL) on BT-474 cell migration was substantially attenuated upon concurrent CLDN19 silencing (**Figures 8C, D**). At the molecular level, the FAT2-induced downregulation of Vimentin was partially reversed by CLDN19 knockdown (**Figures 8B, E**), further substantiating the role of CLDN19 as a functional downstream effector of FAT2 in EMT regulation.

Collectively, these findings demonstrate that FAT2 suppresses breast cancer cell migration and EMT, at least in part, through transcriptional upregulation of the tight junction protein CLDN19.

### 3.5 Consistent co-expression and positive correlation of FAT2 and CLDN19 in clinical breast cancer specimens

**Figure 9:**
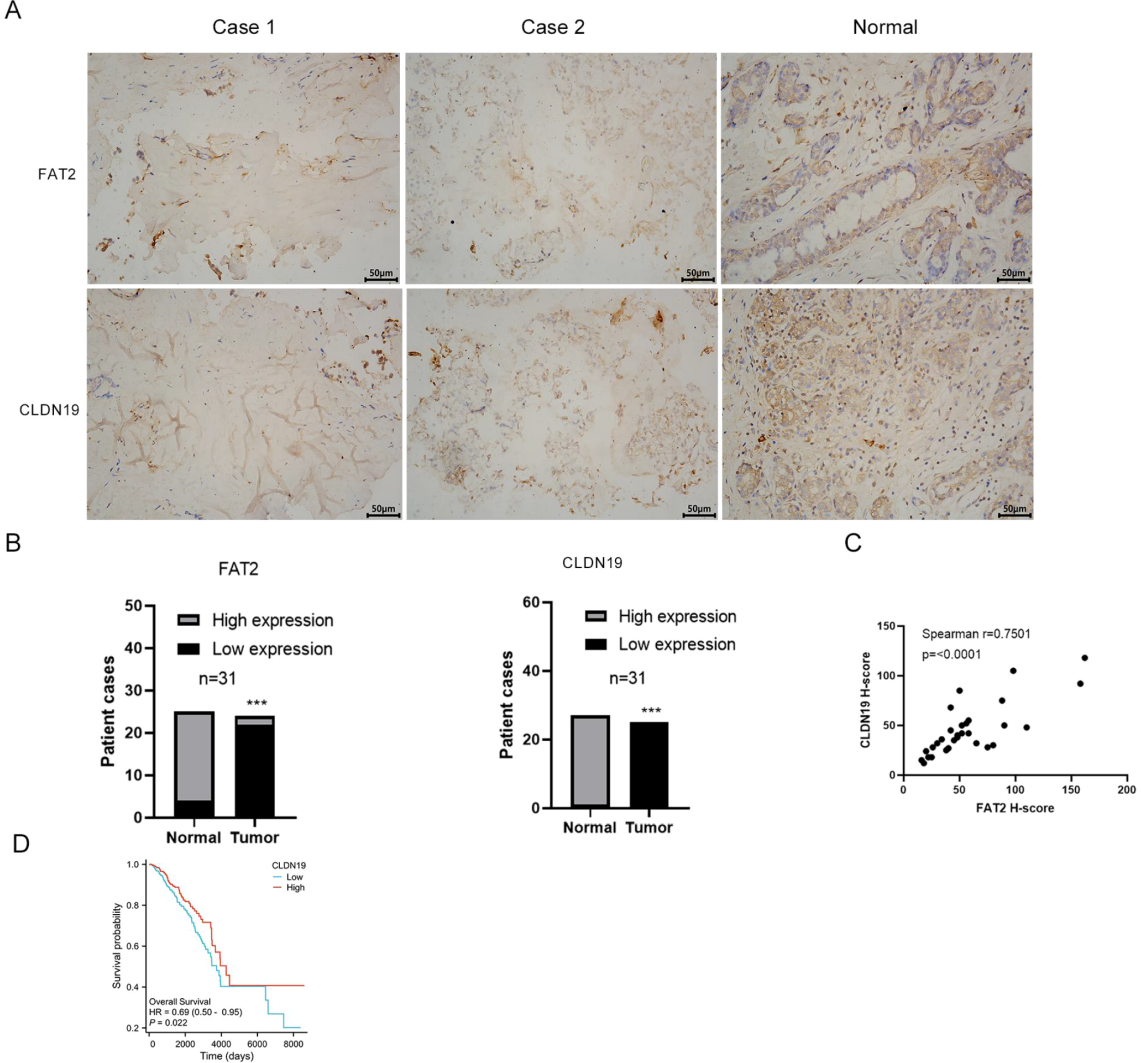
Clinical validation of the FAT2–CLDN19 regulatory axis in breast cancer tissues. **(A, B)** Representative immunohistochemical staining and quantitative analysis of FAT2 **(A)** and CLDN19 **(B)** protein expression in 31 paired breast cancer and adjacent normal tissue samples. **(C)** Spearman correlation analysis showing a significant positive correlation between FAT2 and CLDN19 protein expression in breast cancer tissues (*r* = 0.75, *P* < 0.001). **(D)** Kaplan–Meier survival analysis demonstrating that high CLDN19 mRNA expression is associated with improved overall survival in breast cancer patients (HR = 0.69, *P* = 0.022; TCGA-BRCA cohort).

To validate the *in vitro* findings in a clinical context, immunohistochemical staining for FAT2 and CLDN19 was performed on 31 paired breast cancer and adjacent normal tissue specimens. In agreement with the TCGA data, both FAT2 and CLDN19 protein expression levels were significantly lower in tumor tissues compared with their matched normal counterparts (mean IHC score ± SD: FAT2, 46.7 ± 22 vs. 210 ± 35; CLDN19, 30.5 ± 18 vs. 240 ± 40; both *P* < 0.001; **Figures 9A, B**).

Notably, a strong positive correlation was observed between FAT2 and CLDN19 protein expression across all tumor samples (Spearman’s *r* = 0.75, *P* < 0.001; **Figure 9C**), consistent with the transcriptomic correlation observed in the TCGA dataset. Furthermore, Kaplan–Meier analysis of the TCGA-BRCA cohort demonstrated that high CLDN19 mRNA expression was associated with significantly improved overall survival (HR = 0.69, *P* = 0.022; **Figure 9D**), further underscoring the clinical relevance of the FAT2–CLDN19 axis.

Collectively, these clinical findings corroborate the *in vitro* evidence for a FAT2–CLDN19 regulatory relationship and suggest that the concurrent downregulation of both proteins may contribute to breast cancer progression and unfavorable patient outcomes.

## 4 Discussion

In the present study, we systematically investigated the expression, biological function, and molecular mechanism of FAT2 in breast cancer. The principal findings are as follows: (i) FAT2 is significantly downregulated in breast cancer, with its loss associated with adverse clinicopathological features and poor prognosis; (ii) recombinant His-FAT2 protein is actively internalized by breast cancer cells through an energy-dependent endocytic pathway and specifically suppresses migration, invasion, and EMT at sub-cytotoxic concentrations; and (iii) FAT2 exerts its anti-migratory function, at least in part, through transcriptional upregulation of CLDN19.

Efficient cellular uptake is a prerequisite for the biological activity of exogenous recombinant FAT2. Using a trypsin protection assay (15), we demonstrated that approximately 45% of His-FAT2 was internalized and protected from extracellular proteolysis at 37 °C, whereas the trypsin-resistant signal decreased to below 11% at 4 °C, confirming an active, energy-dependent internalization process (16). Notably, a previous study demonstrated that the recombinant SAC domain of Par-4, a cytoplasmically derived protein expressed in *Escherichia coli*, could induce apoptosis in ovarian cancer cells from the extracellular space through binding to cell-surface GRP78 (17), providing a precedent for the uptake of exogenous recombinant proteins by cancer cells. Although the specific endocytic pathway mediating His-FAT2 internalization was not identified, the observed temperature dependence strongly supports canonical energy-dependent endocytosis.

The anti-migratory effect of FAT2 at sub-cytotoxic concentrations rules out non-specific cytotoxicity and highlights a specific role in the regulation of cell motility. FAT2 reversed the EMT phenotype by upregulating E-cadherin and downregulating N-cadherin, Vimentin, and MMP9, consistent with prior evidence implicating the FAT family in EMT regulation (5, 6, 18). Other FAT cadherins, such as FAT1 and FAT4, modulate EMT through contact inhibition, cell polarity, and Hippo signaling (19, 20), providing an evolutionary framework for the tumor-suppressive function of FAT2.

To the best of our knowledge, this study is the first to identify the FAT2–CLDN19 regulatory axis in breast cancer. FAT2 positively regulates CLDN19 at the transcriptional level, and siRNA-mediated CLDN19 knockdown partially reversed the FAT2-mediated suppression of migration and Vimentin expression, establishing CLDN19 as a functional downstream effector. As a core component of tight junctions, claudins maintain intercellular barrier function and cell polarity (21), and their dysregulation has been closely linked to cancer metastasis (22). CLDN19 has been shown to inhibit breast cancer progression by downregulating UBE2C and attenuating Wnt/β-catenin signaling (9); Accordingly, FAT2 may indirectly suppress this axis through CLDN19 upregulation, ultimately inhibiting EMT and metastasis. Our data position FAT2 upstream of the CLDN19–UBE2C–Wnt cascade, adding a novel dimension to breast cancer metastasis-regulatory networks (23, 24).

Our findings further reveal tissue-specific functional divergence of FAT2. Whereas FAT2 mutations in sweat gland carcinoma, lymphomas, and cholangiocarcinoma represent potential driver alterations (25–27), our data highlight the role of FAT2 as a metastasis suppressor in breast cancer, likely reflecting differences in tissue-specific signaling contexts (28, 29). From a translational perspective, FAT2 shows promise as a prognostic biomarker, although its independent predictive value requires validation in larger patient cohorts (30, 31). The FAT2–CLDN19 axis provides candidate therapeutic targets, and strategies aimed at reactivating this tumor-suppressive axis may represent promising anti-metastatic therapeutic approaches (32–35).

Several limitations of this study should be acknowledged: (1) the precise mechanism by which FAT2 regulates CLDN19 transcription remains to be defined; (2) the link to UBE2C/Wnt signaling is inferred from the published literature rather than directly validated; (3) the specific endocytic pathway and intracellular fate of the internalized FAT2 protein remain to be characterized; (4) the distinction between exogenous protein accumulation and endogenous protein upregulation requires clarification; and (5) validation in animal models and larger independent patient cohorts is essential to confirm the clinical relevance of the FAT2–CLDN19 axis.

## 5 Conclusion

In summary, this study demonstrates that FAT2 is significantly downregulated in breast cancer and functions as a tumor suppressor by inhibiting cell migration, invasion, and EMT through transcriptional upregulation of CLDN19. Recombinant FAT2 protein is internalized by breast cancer cells through an energy-dependent endocytic pathway and exerts anti-migratory effects at sub-cytotoxic concentrations. The concurrent downregulation and positive correlation of FAT2 and CLDN19 were corroborated in clinical tissue specimens. These findings identify the FAT2–CLDN19 axis as a novel regulatory pathway in breast cancer progression that may serve as a prognostic biomarker and therapeutic target for anti-metastatic intervention.

## Data Availability

The datasets presented in this study can be found in online repositories. The names of the repository/repositories and accession number(s) can be found in the article/supplementary material.
